# Acute Myocardial Infarction Complicated by Papillary Muscle Rupture and Cardiogenic Shock Requiring ECMO Support in a Patient with Bipolar Disorder and Chronic Cannabis Use

**DOI:** 10.3390/life16060879

**Published:** 2026-05-24

**Authors:** Oana Elena Branea, Mihaly Veres, Oana Frandeș, Matild Keresztes, Mihai Claudiu Pui, Ciprian Fișcă, Radu Bălău, Leonard Azamfirei

**Affiliations:** 1 Anesthesiology and Intensive Care Department, George Emil Palade University of Medicine, Pharmacy, Science and Technology of Targu Mures, 540142 Targu Mures, Romania; oana.branea@umfst.ro (O.E.B.); leonard.azamfirei@umfst.ro (L.A.); 2 Doctoral School of Medicine and Pharmacy, George Emil Palade University of Medicine, Pharmacy, Science and Technology of Targu Mures, 540142 Targu Mures, Romania; oana.frandes@umfst.ro (O.F.); keresztes.matild.25@stud.umfst.ro (M.K.); mihai.pui@umfst.ro (M.C.P.); 3 Department of Intensive Care, Emergency Institute for Cardiovascular Diseases and Transplantation, 540136 Targu Mures, Romania; ciprianfisca@yahoo.com; 4 Department of Cardiovascular Surgery, George Emil Palade University of Medicine, Pharmacy, Science and Technology of Targu Mures, 540142 Targu Mures, Romania; radu.balau@umfst.ro

**Keywords:** acute myocardial infarction, papillary muscle rupture, cardiogenic shock, coronary bypass, mitral valve replacement, ECMO, Type I Bipolar Disorder, cannabis use

## Abstract

Cardiogenic shock secondary to acute myocardial infarction complicated by mechanical failure remains associated with high mortality despite advances in cardiac surgery and mechanical circulatory support. We report the case of a 42-year-old patient with posterior papillary muscle rupture leading to severe mitral regurgitation, managed with emergency surgical intervention and extracorporeal membrane oxygenation. The patient, with a history of Type I Bipolar Disorder under long-term lithium therapy and chronic Cannabis use, presented in critical condition with cardiogenic shock (Killip IV), acute pulmonary edema, and ST-segment elevation myocardial infarction in the infero-posterior territory. Coronary angiography revealed right coronary artery occlusion and involvement of an obtuse marginal branch. Emergency mitral valve replacement with a mechanical prosthesis and aortocoronary bypass were performed. Due to failure to wean from cardiopulmonary bypass, central veno-arterial ECMO was initiated. The postoperative course was complicated by hemodynamic instability and recurrent pericardial collections requiring repeated surgical interventions and conversion to peripheral ECMO. Multiorgan dysfunction developed, including hepato-renal failure requiring hemofiltration, neurological injury, respiratory impairment, and neuropsychiatric complications. Despite these challenges, progressive recovery was achieved under intensive multidisciplinary management. This case emphasizes the importance of early surgical correction and tailored ECMO support in managing post-infarction mechanical complications.

## 1. Introduction

### 1.1. Clinical Context: AMI-CS and Mechanical Complications

Cardiogenic shock (CS) is characterized by systemic hypoperfusion and tissue hypoxia resulting from cardiac dysfunction [1,2]. According to Shock Academic Research Consortium, SHARC criteria define CS and involve a systolic blood pressure below 90 mm Hg for longer than 30 min or the need for inotropic, vasopressor, or mechanical circulatory support to preserve blood pressure, together with signs of systemic hypoperfusion [3]. Its most frequent cause is acute myocardial infarction (AMI), with subsequent myocardial necrosis and impaired ventricular function [2]. In addition, papillary muscle rupture is a rare (occurs in 1% to 3% of patients [4]) but catastrophic complication of AMI in which CS is primarily driven by acute severe mitral regurgitation, making time to surgery the most important modifiable determinant of outcome [5]. Still, over the past decade, improvements in therapy have not translated into better outcomes, as the incidence of AMI-CS has increased and mortality continues to approach 50% [1,2,5].

### 1.2. Emerging Etiology: Cannabis—An Observational Perspective

From a less typical perspective, the expanding worldwide use of Cannabis has coincided with a growing body of reports linking its consumption to AMI [6,7,8]. This association has been explained through several proposed mechanisms. The cardiovascular effects of Cannabis are largely mediated by Δ9-tetrahydrocannabinol (THC), acting mainly on the CB1 receptor and secondarily on the CB2 receptor within the endocannabinoid system [9]. A central mechanism is the imbalance between myocardial oxygen consumption and supply. THC-induced tachycardia increases oxygen demand, whereas smoking reduces oxygen availability [8,9]. Endothelial impairment further contributes by facilitating coronary vasospasm. In addition, Cannabis may promote platelet activation and thrombus formation, even in structurally normal coronary arteries [9]. Arrhythmic events may further aggravate myocardial ischemia [8,9]. Clinical reports typically describe young patients with few conventional risk factors, presenting shortly after Cannabis exposure. While causality remains difficult to establish, the consistent temporal association and mechanistic plausibility indicate that Cannabis, can precipitate acute myocardial ischemia [6,7,8]. Notably, while most reported cases involve transient or less complicated ischemic events, severe outcomes such as mechanical cardiac rupture are rarely encountered. In our case, the association between cannabis use and myocardial infarction remains observational and should be interpreted with caution. Cannabis exposure may have contributed to the clinical presentation, but a direct causal relationship cannot be established. Further studies are needed to better delineate the cardiovascular effects of cannabis use in young patients.

### 1.3. Management Challenges

Management of CS caused by mechanical complications demands immediate recognition and coordinated intervention [1,10]. Advanced circulatory support, including extracorporeal membrane oxygenation (ECMO), can stabilize patients as a bridge to surgery or to recovery [11,12]. Clinical decision-making becomes more challenging when overlapping factors such as substance use or psychiatric comorbidities are present, often delaying presentation or complicating management [8,11,12]. Despite increasing reports of Cannabis-associated myocardial infarction, data remain limited on progression to mechanical complications requiring extracorporeal support, underscoring an important gap in current evidence and clinical guidance.

### 1.4. Particularity of the Case

This case brings together several uncommon features in a young patient with cardiovascular risk factors, such as tobacco use, stage II blood hypertension, and mixed dyslipidemia. In additional to these factors, the AMI temporally linked to Cannabis use, could suggest a non-atherosclerotic cause and substance exposure may have contributed to the onset. The coexistence of Type I Bipolar Disorder added complexity, given its association with substance use and potential challenges in clinical management. An important particularity was the early development of a severe mechanical complication. Papillary muscle rupture occurrence pointed to an extensive, severe ischemic insult. The resulting acute mitral regurgitation led to rapid hemodynamic deterioration, with pulmonary congestion and reduced cardiac output. The progression to refractory cardiogenic shock further emphasized the severity of the presentation, limiting the response to standard medical therapy. Urgent surgery included coronary bypass and mechanical mitral valve replacement. Persistent failure to wean from cardiopulmonary bypass led to central veno-arterial ECMO. Although the postoperative period was complicated by instability and recurrent pericardial effusions necessitating repeated interventions and transition to peripheral ECMO, the patient had a good outcome under close multidisciplinary care.

AMI complicated by papillary muscle rupture, requiring combined surgical revascularization, mechanical mitral valve replacement, and ECMO support, has been infrequently reported, particularly in young individuals known for cannabis use. This case further expands the currently limited evidence on the potential for severe cardiovascular effects linked to Cannabis use.

## 2. Case Presentation

**Figure 1:**
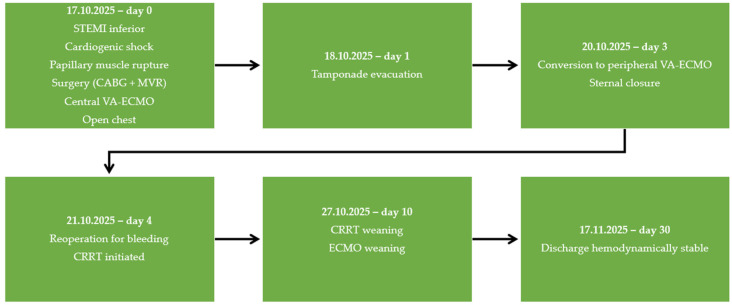
Timeline of the patient’s presentation, surgical management, and intensive care course. CABG—coronary artery bypass grafting; MVR—mitral valve replacement; VA-ECMO—veno-arterial extracorporeal membrane oxygenation; ECMO—extracorporeal membrane oxygenation; CRRT—continuous renal replacement therapy; STEMI—ST-segment elevation myocardial infarction.

We report a rare case of a 42-year-old male with a history of Type I Bipolar Disorder and chronic Cannabis use, who was admitted in a cardiac ICU presenting a critical condition displaying cardiogenic shock and acute pulmonary edema related to ischemic myocardial infarction and severe valvular dysfunction, subsequently confirmed as mitral valve rupture. Figure 1 highlights the chronological sequence of major clinical events, detailing the emergency surgical procedures, the necessity for mechanical circulatory support, and the postoperative complications encountered, from the initial presentation through intensive care treatment and subsequent recovery leading to discharge.

### 2.1. Patient’s History, Disease Onset, and Clinical Examination

A patient with significant cardiovascular risk factors, including chronic tobacco use, stage II hypertension, and mixed dyslipidemia, as well as a psychiatric history of Type I Bipolar Disorder characterized by manic and psychotic episodes alongside harmful Cannabis use, presented urgently at the hospital on 17 October 2025. The expressed symptoms were retrosternal anginal chest pain, dyspnea progressing to orthopnea, productive cough, and profuse sweating. At the time of admission, the patient’s condition was critical, with clinical and hemodynamic features indicative of cardiogenic shock and acute pulmonary edema. The severity of presentation corresponded to Killip class IV. Clinical examination identified regular cardiac rhythm with preserved heart sounds and a grade IV/VI systolic murmur auscultated at the mitral area. No evidence of peripheral edema was noted upon presentation.

### 2.2. Initial Work-Up and Intervention

**Figure 2:**
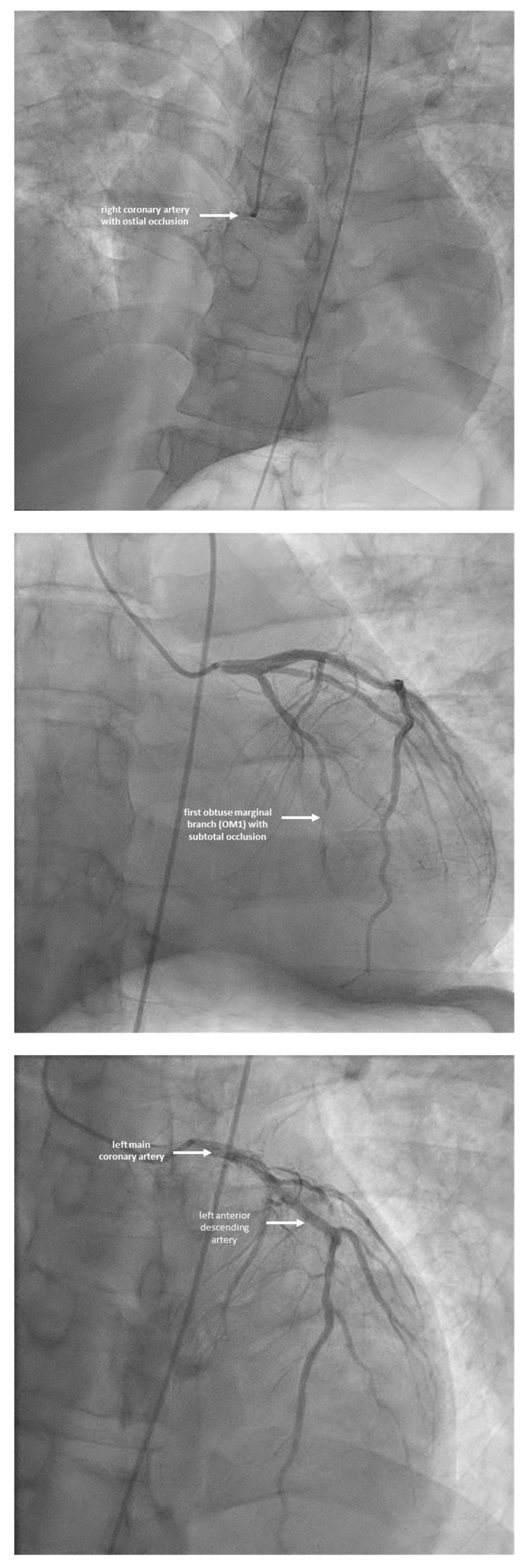
Coronary angiographic findings demonstrating right coronary artery with ostial occlusion and obtuse marginal branch subocclusion; No critical stenosis on the left coronary system.

Initial work-up revealed markedly elevated cardiac biomarkers, with high-sensitivity troponin I reaching 20,550 pg/mL and CK-MB 294.37 ng/mL, consistent with extensive myocardial injury. These findings correlated with electrocardiographic changes, which demonstrated sinus rhythm with ST-segment elevation in leads DII, DIII, and aVF, along with reciprocal ST depression in leads V2–V4 and pathological inferior Q waves, supporting the diagnosis of acute infero-posterior myocardial infarction. The echocardiographic assessment demonstrated significant mitral regurgitation attributed to the rupture of the posterior papillary muscle, with a left ventricular ejection fraction of approximately 30% perioperativly. Urgent coronary angiography confirmed a right-dominant system, demonstrating ostial occlusion of the right coronary artery and a significant subocclusion of an obtuse marginal branch, with impaired distal coronary flow. The left coronary system showed no critical stenosis (Figure 2).

Given the presence of acute mechanical complication of papillary muscle rupture resulting in severe mitral regurgitation and cardiogenic shock, percutaneous coronary intervention was not attempted. The patient was referred directly for emergency cardiac surgery. This procedure involved aortocoronary bypass using a saphenous vein graft to the posterior interventricular artery and replacement of the mitral valve with a mechanical prosthesis under cardiopulmonary bypass. Severe intraoperative hemodynamic instability prevented weaning from extracorporeal circulation, necessitating initiation of central veno-arterial ECMO (aortic to right atrial cannulation). In light of the critical status and requirement for ongoing support, the sternum was left open to enable close monitoring and facilitate prompt re-intervention if needed.

### 2.3. Critical Postoperative Period

The immediate postoperative course was marked by persistent hemodynamic instability, further complicated by cardiac tamponade secondary to a significant pericardial collection. This required urgent surgical reintervention on 18 October 2025, consisting of evacuation of the collection and pericardial lavage. Despite initial stabilization, ongoing circulatory support remained necessary. On 20 October 2025, central ECMO was discontinued and transitioned to peripheral veno-arterial ECMO via the left femoral vessels, with venous drainage through the right internal jugular vein; sternal closure was performed during the same procedure. The following day, on 21 October 2025, the patient required a second reintervention for recurrent pericardial collection evacuation and surgical hemostasis, along with placement of a left femoral dialysis catheter. Progressive cardiovascular improvement allowed for gradual weaning, and peripheral veno-arterio-venous ECMO support was successfully discontinued on 27 October 2025.

**Figure 3:**
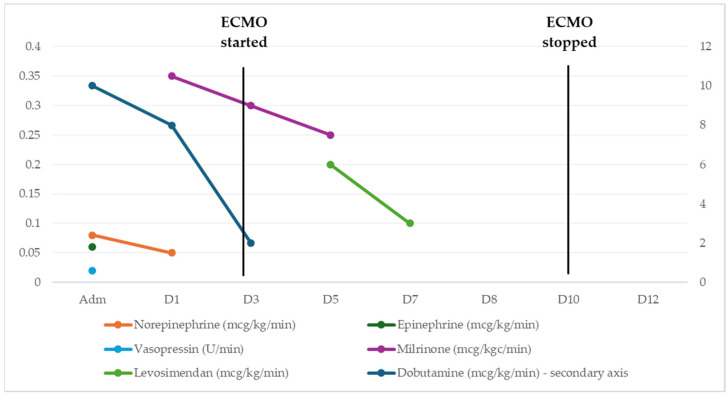
Dynamics of vasoactive and inotropic therapy during ECMO support and weaning.

Moreover, at presentation, the patient required high-dose, multi-agent support, including norepinephrine, epinephrine, vasopressin, and dobutamine, reflecting profound cardiogenic shock. With the initiation of ECMO, a rapid decrease in vasopressor requirements was observed, particularly for norepinephrine and vasopressin, indicating improved circulatory stability and organ perfusion. Inotropic support with dobutamine and milrinone was maintained during the initial ECMO phase, reflecting ongoing myocardial impairment despite mechanical circulatory assistance. A gradual reduction in these agents over time suggested progressive myocardial recovery. Following ECMO withdrawal, pharmacological support was further reduced, with transition to lower-dose therapy and adjunctive agents such as levosimendan. Figure 3 illustrates these trends that reflect a progressive decline in pharmacological support requirements, corresponding to improved hemodynamic status and recovery from severe cardiogenic shock under ECMO.

Furthermore, close monitoring of oxygenation, ventilation, and tissue perfusion was important for maintaining organ function. As presented in Table 1, the combined analysis of gas exchange, perfusion, and acid–base parameters provided a coherent picture of the patient’s pathophysiological evolution during the intensive care hospitalization. At admission, impaired oxygenation (PaO_2_/FiO_2_ 194) coexisted with marked hyperlactatemia (7.12 mmol/L) and metabolic acidosis (pH 7.32, BE −6.2 mmol/L), indicating shock with global tissue hypoperfusion. The elevated Pv–aCO_2_ gap (8 mmHg) and relatively reduced SvO_2_ (62%) further supported inadequate cardiac output and increased oxygen extraction. Following initiation of ECMO, rapid improvement was observed across all domains. Oxygenation normalized (PaO_2_/FiO_2_ > 380), lactate levels decreased dramatically (<1 mmol/L), and acid–base status corrected (pH 7.42–7.45). Concurrent normalization of SvO_2_ and reduction in Pv–aCO_2_ indicated restoration of effective systemic perfusion. After ECMO weaning, oxygenation further improved (PaO_2_/FiO_2_ ~460–490) with reduced FiO_2_ requirements, and perfusion parameters remained stable. Mild acid–base variations and slight lactate increases likely reflect recovery-phase metabolic demand rather than recurrent shock. At discharge, despite lower PaO_2_ values, SvO_2_ remained acceptable and lactate only moderately elevated, indicating stable hemodynamics with residual metabolic stress. Overall, the parallel improvement in oxygenation, perfusion, and metabolic markers reflected progressive recovery from severe cardiogenic shock.

During hospitalization, biochemical trends illustrated the progression of multiorgan dysfunction in the setting of cardiogenic shock and its subsequent resolution under intensive care, including the use of continuous renal replacement therapy (CRRT). Initially, elevated creatinine and urea values indicated acute kidney injury, while high transaminases and LDH levels suggested ischemic hepatic damage. Low albumin reflected the inflammatory state. Despite early partial improvement, sustained metabolic disturbances required CRRT initiation to optimize fluid and solute balance. During extracorporeal support, a marked decline in liver enzymes was observed, indicating improved perfusion. With hemodynamic stabilization, renal function progressively improved, allowing CRRT discontinuation. By discharge, near-normal biochemical values confirmed recovery of both renal and hepatic function. As depicted in Table 2, the laboratory profile reflected the dynamic evolution of hepato-renal function during the intensive care course, consistent with initial multiorgan dysfunction followed by gradual recovery.

Additionally, thoraco-abdomino-pelvic imaging revealed postoperative changes after myocardial revascularization and mitral valve replacement, along with bilateral bronchopneumonia, pleural effusions, pneumopericardium, pneumomediastinum, and subcutaneous emphysema. No imaging evidence of pulmonary thromboembolism was detected. Moreover, neurological deficits emerged during the intensive care stay, with repeated assessments indicating hypoxic–ischemic encephalopathy and flaccid tetraparesis, more prominent on the right side. Brain imaging identified multiple small bilateral cortico-subcortical ischemic lesions and a hyperdense lesion in the left cerebellar hemisphere, which was monitored over time. The neuropsychiatric evolution was characterized by postoperative delirium and significant psychomotor agitation, necessitating dedicated psychiatric treatment. These manifestations were considered multifactorial, related to critical illness, prolonged sedation, and pre-existing psychiatric disease. All of these mentioned findings showed progressive improvement under treatment.

### 2.4. Clinical Outcome at Discharge

At discharge, the patient was hemodynamically stable, with preserved respiratory function and no symptoms at rest. The electrocardiogram showed sinus rhythm with complete left bundle branch block. Transthoracic echocardiography revealed a left ventricular ejection fraction of approximately 40%, a normally functioning mechanical mitral valve prosthesis without evidence of dysfunction or paravalvular leak, and mild-to-moderate tricuspid regurgitation, in the absence of pericardial effusion. Despite clinical stabilization, residual neurological deficits persisted. The patient presented with psychomotor slowing, dysphonia, and dysphagia for liquids, consistent with sequelae of hypoxic–ischemic injury and prolonged critical illness. These impairments required continued multidisciplinary care, including neurological follow-up, speech therapy, and rehabilitation. At discharge, recommendations included close cardiological and neurological monitoring, as well as continuation of supportive therapies to facilitate recovery and reduce the risk of further complications.

## 3. Discussions

### 3.1. Mechanical Complication of AMI: Papillary Muscle Rupture and Cardiogenic Shock

The complications of AMI are conventionally divided into electrical, mechanical, hemodynamic or ischemic, and inflammatory or late groups [10], comprising arrhythmias like ventricular tachycardia or fibrillation, mechanical complications including papillary muscle rupture, ventricular septal rupture, and free wall rupture, hemodynamic consequences such as CS and acute heart failure, and delayed outcomes including pericarditis, left ventricular aneurysm, and mural thrombus [10,13,14,15]. Free wall rupture, ventricular septal rupture, and papillary muscle rupture are mechanical complications that typically develop during the first week following ST-elevation MI, commonly around days 3–5, and are observed in less than 1% of patients in recent series [16,17].

In our case, the initial presentation was marked by profound hemodynamic instability, with the patient admitted in CS and acute pulmonary edema, corresponding to Killip class IV. The underlying mechanism was acute rupture of the posterior papillary muscle, leading to severe mitral regurgitation, and rapid progression to respiratory failure. Although papillary muscle rupture is more commonly described in male older patients [18], our case illustrated its occurrence in a younger individual, aged 42 years, suggesting that additional factors may modulate the risk. Delayed presentation, potentially influenced by coexisting Type I Bipolar Disorder and Cannabis use, may have contributed to the extent of myocardial injury [19,20]. The presence of an infero-posterior infarction was supported by electrocardiographic findings and elevated cardiac biomarkers, consistent with established predictors of mechanical complications. This case highlighted the importance of prompt diagnostic evaluation, particularly through urgent echocardiography and coronarography [21,22,23], which were essential for identifying AMI, acute severe mitral regurgitation and confirming structural complications. Early hemodynamic assessment and coordinated multidisciplinary management were critical in guiding timely therapeutic decisions and improving clinical outcomes.

### 3.2. Cannabis-Associated Myocardial Infarction: Plausible Mechanisms and Limits of Causality

The association between Cannabis use and myocardial infarction has been reported, but remains incompletely understood [6,7]. Despite of this, recent publications emphasized prognostic implications in young patients with acute coronary syndromes rather than incident myocardial infarction in the general population, highlighting Cannabis use as a relevant factor [24,25]. Data from recent cohorts or meta-analyses showed that Cannabis exposure at presentation correlates with an increase in major adverse cardiovascular events during follow-up, pointing to an adverse clinical course [24,25,26]. Suggested mechanisms include sympathetic overactivity [8,9] with increased heart rate and blood pressure, heightened oxygen demand, coronary vasospasm, and platelet activation [27,28]. Evidence is predominantly based on observational data and case reports, with multiple confounders such as tobacco use and other risk factors [29,30,31].

In the present case, the close temporal association between recent Cannabis exposure and symptom onset suggested a potential contributory role. The patient’s young age added to the cardiovascular risk factors (dyslipidemia and stage II hypertension), may support the consideration of an external precipitating factor, while the clinical profile may aid in distinguishing between plaque rupture and vasospastic mechanisms. Nonetheless, the existing evidence is predominantly observational and case-based, with significant confounding from concomitant drugs (lithium treatment) and underlying risk factors (Type I Bipolar Disorder). Importantly, no direct causal link has been established between Cannabis use and mechanical complications such as papillary muscle rupture.

### 3.3. Management of AMI-CS

Published evidence is largely limited to isolated case reports describing mechanical complications after AMI, particularly papillary muscle rupture, typically managed with mitral valve replacement and coronary revascularization, as illustrated by reports written by Mary DA et al. [32], Kyo S et al. [33], Jayawardena S et al. [34], Kamada T et al. [35], Singam NSV et al. [36]. As emphasized in the mentioned case-reports and stated by current European Society of Cardiology and American Heart Association guidelines, the present case illustrated the need for rapid and dynamic management in AMI-CS. Following initial resuscitative measures, urgent coronarography was performed to delineate the underlying coronary pathology, enabling targeted surgical treatment. In the setting of papillary muscle rupture, the patient underwent combined coronary artery bypass grafting and mechanical mitral valve replacement. Due to persistent refractory shock, central ECMO was instituted early to provide circulatory support. The clinical evolution was complicated by the development of cardiac tamponade, necessitating prompt reoperation. In this context, support was subsequently converted to peripheral veno-arterial ECMO, allowing continued hemodynamic stabilization. Despite multiple critical events, the structured escalation of therapy and multidisciplinary approach contributed to a favorable hemodynamic recovery.

### 3.4. Implications for Future Practice

Management required close coordination across specialties in a setting of AMI-CS complicated by papillary muscle rupture. The rapid progression to mechanical failure underscores the importance of early recognition and prompt imaging. The optimal timing of ECMO remains uncertain, but this case supports expanding dedicated ECMO teams across different healthcare settings to ensure timely initiation, safe transfer, and consistent protocols. Moreover, there are reviews that summarize current treatment strategies, focusing on revascularization, mechanical circulatory support, emerging drug therapies to guide tailored AMI-CS management [1,12,16,37,38], while a personalized strategy that incorporates clinical, hemodynamic, and behavioral data may be associated with improved outcomes [39,40,41].

The possible association with Cannabis highlights the relevance of non-traditional triggers of myocardial infarction, particularly in patients without significant atherosclerotic burden. Careful evaluation of substance use, psychiatric comorbidities, and treatment adherence is essential. Within a personalized medicine framework, these patient-specific factors may influence both risk assessment and therapeutic decisions [39], especially in complex presentations involving mechanical complications.

### 3.5. Key Learning Points

The case highlights how a rare post-infarction mechanical complication can rapidly lead to cardiogenic shock. Papillary muscle rupture resulted in abrupt hemodynamic collapse driven by severe mitral regurgitation rather than primary ventricular failure. Cannabis exposure may have contributed to the initial ischemic event through several proposed mechanisms, like mediated by vasospasm or sympathetic activation, though evidence is based only on observation. Prompt recognition and rapid multidisciplinary management, including ECMO and surgery, were essential to patient recovery.

## 4. Conclusions

AMI complicated by papillary muscle rupture and CS demands immediate recognition and tailored management. In this patient, surgical repair combined with ECMO enabled stabilization despite severe complications. Individual factors, including psychiatric comorbidity and Cannabis exposure, highlight the need for a personalized approach. Multidisciplinary care remains essential for favorable outcomes.

## Figures and Tables

**Table 1:** Dynamic changes in oxygenation, perfusion, and acid–base status during intensive care course.

	T0	Day 1	Day 3	Day 5	Day 7	Day 8	Day 10	Day 12	Day 14	Discharge
pH	7.32	7.42	7.45	7.43	7.4	7.42	7.35	7.34	7.44	7.34
PaO_2_ (mmHg)	136	191	96.2	162	99.8	111	98	97.7	92	55.6
PaCO_2_ (mmHg)	37	44.3	40.5	40	38.9	39.3	44	41.3	42.5	39.2
Pv–a CO_2_ (mmHg)	8	6	5	6	5	4	4	5	4	NA
HCO_3_^−^ (mmol/l)	28.8	27.4	25.9	25.7	21.3	25	23	22.5	22.4	20.9
BE	−6.2	3.8	1.8	1.7	−3.8	0.8	2.7	−0.8	−0.5	−2.2
SaO_2_ (%)	99.6	99.4	97.1	99.2	97.9	98.3	97.6	97	98	NA
SvO_2_ (%)	62	70	72	70	68	65	64.8	72.5	70	68.9
FiO_2_ (%)	0.7	0.5	0.4	0.4	0.5	0.4	0.2	0.2	0.2	NA
PaO_2_/ FiO_2_	194.2	382	240.5	405	199.6	277.5	490	488.5	460	NA
Lactate (mmol/l)	7.12	0.73	1.18	0.83	0.73	1.03	1.4	1.3	1.44	2.34

T0—admission; PaO_2_—partial pressure of arterial oxygen; PaCO_2_—partial pressure of arterial carbon dioxide; Pv–aCO_2_—veno-arterial carbon dioxide partial pressure difference; HCO_3_^−^—bicarbonate; BE—base excess; SaO_2_—arterial oxygen saturation; SvO_2_—mixed venous oxygen saturation; FiO_2_—fraction of inspired oxygen; PaO_2_/FiO_2_ ratio—ratio of arterial oxygen partial pressure to fraction of inspired oxygen; NA—not available.

**Table 2:** Dynamics of hepato-renal function markers during intensive care course.

	T0	Day 1	Day 3	Day 5	Day 7	Day 8	Day 10	Day 12	Day 14	Discharge
Creatinine (mg/dL)	2.22	1.81	1.7	1.64	1.76	1.09	1.26	1.37	1.25	1.2
Urea (mg/dL)	64.63	96.73	96.21	84.65	92.19	103.3	70.2	66.7	54	35
AST (U/L)	576	271	164	350	650	128	91	43	40	25
ALT (U/L)	436	135	60	151	279	205	119	67	62	20
LDH (U/L)	1568	842	412	476	941	521	466	NA	NA	NA
Albumin (g/dL)	2.9	3.2	2.5	3.4	3.7	3.8	3.9	3.8	NA	4.2

Creatinine—serum creatinine; Urea—blood urea; AST—aspartate aminotransferase; ALT—alanine aminotransferase; LDH—lactate dehydrogenase; Albumin—serum albumin; NA—not available.

## Data Availability

All data, including electronic records, are available in the archives of the County Emergency Clinical Hospital of Targu Mures, specifically at the Emergency Institute for Cardiovascular Diseases and Transplantation, Targu Mures, Romania.

## Abbreviations

The following abbreviations are used in this manuscript:

APACHE II	Acute Physiology and Chronic Health Evaluation II
AST	aspartate aminotransferase
BE	Base Excess
CABG	Coronary Artery Bypass Grafting
CAR	C-reactive protein to albumin ratio
CRP	C-reactive protein
CRRT	Continuous Renal Replacement Therapy
ECMO	Extracorporeal Membrane Oxygenation
FiO_2_	fraction of inspired oxygen
HCO_3_^−^	bicarbonate
hs-cTn	high-sensitivity cardiac troponin
ICU	Intensive Care Unit
LDH	lactate dehydrogenase
MVR	mitral valve replacement
NA	not available
NLR	neutrophil-to-lymphocyte ratio
NT-proBNP	N-terminal pro-B-type natriuretic peptide
PaCO_2_	partial pressure of arterial carbon dioxide
PaO_2_	partial pressure of arterial oxygen
PaO_2_/FiO_2_	arterial oxygen partial pressure to inspired oxygen fraction ratio
PCT	procalcitonin
Pv–aCO_2_	veno-arterial carbon dioxide partial pressure difference
SaO_2_	arterial oxygen saturation
SAPS	Simplified Acute Physiology Score
SII	Systemic Immune-Inflammation Index
SOFA	Sequential Organ Failure Assessment
STEMI	ST-segment elevation myocardial infarction
SvO_2_	mixed venous oxygen saturation
T0	admission
VA-ECMO	Veno-Arterial Extracorporeal Membrane Oxygenation
